# Effects of low and moderate treadmill exercise on liver of d‐galactose‐exposed aging rat model

**DOI:** 10.14814/phy2.14279

**Published:** 2019-11-13

**Authors:** Widya Wasityastuti, Nurfatma A. Habib, Dwi C. R. Sari, Nur Arfian

**Affiliations:** ^1^ Department of Physiology Faculty of Medicine Public Health and Nursing Universitas Gadjah Mada Yogyakarta Indonesia; ^2^ Master in Biomedical Sciences Faculty of Medicine Public Health and Nursing Universitas Gadjah Mada Yogyakarta Indonesia; ^3^ Department of Anatomy Faculty of Medicine Public Health and Nursing Universitas Gadjah Mada Yogyakarta Indonesia

**Keywords:** Aging, d‐galactose, exercise, liver fibrosis, M1/M2 ratio

## Abstract

Aging increases liver susceptibility to diseases and it causes inflammation in liver tissue which can lead to fibrosis. Studies suggest that aging is caused by the accumulation of free radicals. Lack of physical activity can lower hormone levels and increase free radicals that can accelerate the aging process. Hence, physical activity is very important to maintain functions of organs. This research was aimed to study the effects of low and moderate treadmill exercise on d‐Galactose‐exposed aging rat model by evaluating the degree of hepatic fibrosis, number of M1 and M2, and M1/M2 ratio. Twenty‐four 3‐month‐old male Wistar aging model rats were randomly divided into four groups, that is, three treatment groups with daily 300 mg kgBW^−1^ d‐Galactose injection administrated intraperitoneally for 4 weeks and 1 control group with normal saline injection. Two of the d‐Galactose treated groups were given low and moderate treadmill exercise for 4 weeks. It was concluded that low intensity treadmill exercise significantly lowered the degree of d‐Galactose‐exposed hepatic fibrosis, and moderate treadmill exercise was able to restore the injured liver tissue back to the non‐aging state. Administration of d‐Galactose causes inflammation marked by the elevated number of M1 and M2 macrophages. Moderate treadmill exercise drove M1/M2 ratio back to the control condition.

## Graphical abstract

We investigated the potential hepatoprotective effects of treadmill exercise in d‐Galactose‐exposed ageing rat model. Administration of d‐Galactose caused liver fibrosis and inflammation marked by the elevated number of M1 and M2 macrophages. Low and moderate treadmill exercise could significantly lower the degree of hepatic fibrosis and restored the M1/M2 ratio back to the non‐aging state.

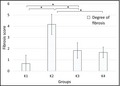

## Introduction

Aging is a decline in fitness components due to a progressive deterioration in body functions and the loss of the ability to maintain homeostasis (Ko et al. 2013). The ability of tissues to repair themselves and maintain their normal structure and function decreases with age; therefore, old people are more susceptible to diseases such as chronic liver disease (Kim et al. 2015). Chronic liver inflammation leads to liver fibrosis or even cirrhosis which causes more than 1 million deaths each year worldwide (Mokdad et al. 2014; Kayama & Brenner 2017).

Liver is an organ with many vital functions. However, liver function decreases with age (Okudan and Belviranli 2016). In the liver, there are macrophages producing inflammatory (M1) and anti‐inflammatory (M2) cytokines that play important roles in innate immunity. Both subtypes of liver macrophages can change form from one to another so that they can secrete pro‐inflammatory and anti‐inflammatory cytokines simultaneously. Aging affects many cellular processes including macrophage polarization (Mahbub et al. 2012). In mouse enteric nervous system, aging promotes switching of M2 to M1 (Becker et al. 2018). However, in fibrotic liver, M2 macrophages are reported to be more predominant and they exhibit hepatoprotective effect against fibrosis by preventing hepatocyte apoptosis (Bai et al. 2017; Amer et al. 2019). Physical activity is also known to influence macrophage polarization. Moderate intensity exercise in sedentary females accelerates phenotype switching from M1 to M2 thus lowering M1/M2 ratio (Ruffino et al. 2016). On the contrary, other studies reported that moderate exercise in healthy males increases the production of Th1 cytokines which are involved in M1 activation (Zamani et al. 2017). In general, macrophage polarization shows aberrant fashion depending on its microenvironment and is affected by pathophysiological condition (Wang et al. 2014).

Physical activity is very important to improve the function of organs in elderly people, such as increasing neurogenesis, angiotensin, and blood flow to the brain (Dimitrijevic et al. 2015). Linden et al. (2016) stated that moderate aerobic exercise can lower non‐alcoholic steatohepatitis (NASH) and hepatic fibrosis in rats. In addition, aerobic exercise also decreases systemic inflammation in elderly people by enhancing antioxidant defense in the body (Soto et al. 2015; He et al. 2016). Fully functioning antioxidant defense system helps the body to remove reactive oxygen species (ROS) (Nunes‐Silva & Freitas Lima 2014).

d‐Galactose has been used in several studies to accelerate aging. d‐galactose exposure can lead to an increase in MDA causing oxidative stress in various tissues (Ho et al. 2003; Huang et al. 2013). d‐Galactose also contributes to the production of ROS and reacts with amino acids to form advanced glycation en d‐products (AGE) which also occur in normal aging, particularly in the liver and brain (Parameshwaran et al. 2010; Ji et al. 2017). The present study aimed to investigate the effect of low and moderate treadmill exercise on liver of d‐Galactose‐exposed aging rat model. The present study focusing on liver fibrosis, number of M1 and M2, and M1/M2 ratio was an extension of a previous study by Partadiredja et al. (2018).

## Methods

### Ethical approval

The experimental protocol was performed in accordance with guidelines established by the Ministry of Research, Technology, and Higher Education and was approved by the Animal Ethics Committee of *Lembaga Pusat Penelitian Terpadu (LPPT) Universitas Gadjah Mada* with reference number 00055/04/LPPT/VI/2017 on 05 June 2017.

### Animals and d‐galactose treatment

This study used 24 male Wistar rats weighing 200–300 g which were obtained from the animal houses of Universitas Islam Indonesia, the Faculty of Pharmacy, and the Department of Pharmacology and Therapy, Faculty of Medicine, Public Health and Nursing, Universitas Gadjah Mada. The rats were acclimatized at controlled room temperature with 12‐h light‐dark cycle for 7 days prior to the experiment and ad libitum access to water and food. The rats were randomly divided into four groups: K1 (negative control: without d‐Galactose exposure but given intraperitoneal saline injection and no exercise), K2 (positive control: given d‐Galactose exposure and no exercise), K3 (given d‐Galactose exposure and low intensity exercise), and K4 (given d‐Galactose exposure and moderate intensity exercise). d‐Galactose in 0.9% NaCl (Tokyo Chemical Industries, Japan) was given as a dose of 300 mg kgBW^−1^ and was injected daily to all rats in K2, K3, and K4 groups for 4 weeks.

### Treadmill exercise protocol

Treadmill adaptation was given to all rats with d‐Galactose exposure for 3–7 days using a modified Brown et al. (2007) protocol on treadmill apparatus (Gama Tread version 2010, Faculty of Medicine, Public Health and Nursing, Universitas Gadjah Mada). During the adaptation period, the rats were introduced to run on the treadmill at the slowest speed (11 m min^−1^) with 0° slope. Rats which were reluctant to run on the adaptation period were put in K2 group. Later on, the maximum speeds of each rat in K3 and K4 groups were measured to estimate the VO_2_max index. VO_2_max index was calculated using the following formula:

$${\text{VO}}_{2}\max\text{Index}=\left[\text{maximum speed (m}\;{\text{min}}^{-1})\right]\;\times \;\left[\text{slope}\;\left(\%\right)\times 100\right]\;\times \;\left[\text{BW (kg)}\right].$$

Slope of 0° equals to 1 in the equation. Afterward, the rats in K3 and K4 groups were given treadmill exercise at the speed of 45% (low intensity) and 55% (moderate intensity) of the previously determined individual VO_2_max Index, respectively.

The treadmill exercise was given four times a week for 4 weeks. Each exercise was conducted after 9 am, and the slope was maintained at 0° during exercise. The exercise started with 5 min of warming‐up period at 20% of maximum speed, 20 min of main exercise at low (K3) or moderate (K4) intensity, followed by 5 min of cooling‐down period at 20% of maximum speed.

### Tissue collection

Tissue collection was performed at the end of the fourth week of treadmill exercise. All rats were anesthetized using ketamine HCl 40 mg/kgBW (PT Guardian Pharmatama, Jakarta, Indonesia) and euthanized. Right lobe of the liver was harvested and put in fixative (10% neutral buffered formalin) for 24 h.

### Hepatic fibrosis examination using picro‐sirius red staining method

Liver tissues were processed for histological examination using paraffin method. The tissues were dehydrated using graded alcohol and then cleared in xylene. Paraffin infiltration and tissue embedding in paraffin wax were performed afterwards. The tissues were then sectioned with a thickness of 4 *μ*m and placed on deck glasses for picro‐sirius red staining. The histological slides were stained with Weigert’s hematoxylin to dye the nucleus and stained with picro‐sirius red for 1 h to dye the collagen fibres. The histological slides were then mounted and ready for microscopic observation. Fifteen random fields of view were observed from each sample and photos were taken. Fibrosis score ranging from 0 to 6 was assigned to each photo and data were documented. The fibrosis score was determined using Ishak system (Standish et al. 2006) consisting of 0‐6 stages with the following criteria: 0: No fibrosis (normal); 1: Fibrous expansion of some portal areas ± short fibrous septa; 2: Fibrous expansion of most portal areas ± short fibrous septa; 3: Fibrous expansion of most portal areas with occasional portal to portal (P‐P) bridging; 4: Fibrous expansion of portal areas with marked bridging P‐P as well as portal to central (P‐C); 5: Marked bridging (P‐P and/or P‐C) with occasional nodules (incomplete cirrhosis); and 6: Cirrhosis, probable or definite.

### Immunohistochemical examination

Sectioned liver tissues with thickness of 4 *μ*m on coated deck glasses were stained against CD68 (*Abcam Ab955*) and arginase‐1 (*Santa Cruz Biotechnology Cat# sc‐20150,* *RRID* *:* *AB_2058955*) antibodies. Antigen retrieval was performed by incubation in citrate buffer solution and heated in a microwave. Blocking peroxidase was conducted with 3% H_2_O_2_ solution in PBS. The slides were then incubated with background sniper solution. The slides were examined using light microscope and photos of 15 random fields of view for each slide were taken with Optilab software using 400× magnification. Cell count was performed using ImageJ software.

### Statistical analysis

Degree of fibrosis data were not normally distributed, Kruskal–Wallis test was used consequently. Mann‐Whitney post hoc test was performed to see the differences between groups.

Number of M1 data were normally distributed after data transformation, therefore number of M2, M1/M2 ratio, and transformed number of M1 were analyzed using one‐way ANOVA followed by post hoc test to see significant differences between groups.

## Results

**Figure 1:**
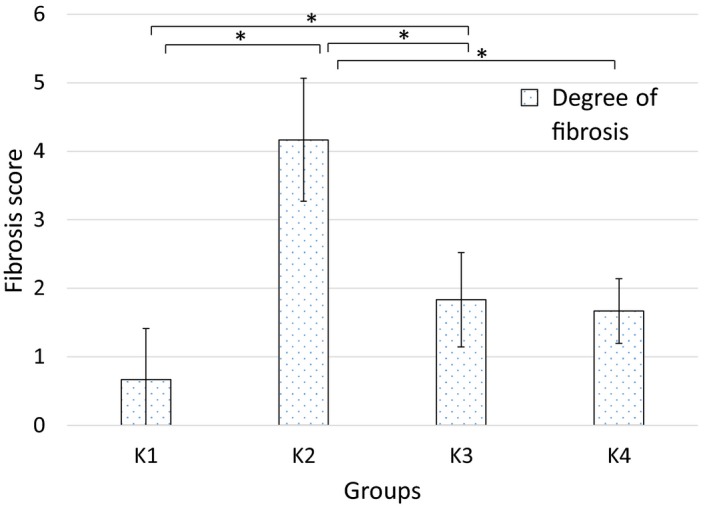
Means (SD) of hepatic fibrosis degree between study groups. K1, 0.9% NaCl intraperitoneal (ip); K2, 300 mg/mL/kg BW d‐Galactose (ip); K3, 300 mg/mL/kg BW d‐Galactose (ip) + low intensity treadmill exercise; K4, 300 mg/mL/kg BW d‐Galactose (ip) + moderate intensity treadmill exercise; * statistically significant between 2 treatment groups.

Figure 1 showed the degree of fibrosis in all groups. d‐Galactose treated group (K2) demonstrated hepatic fibrosis as shown by the significant higher degree of fibrosis compared to control (K1) group (*P* = 0.002). Kruskal–Wallis analysis of these data revealed significant differences among groups. Exercise‐treated groups (K3 and K4) showed significant lower degree of fibrosis compared to K2 group with *p* values of 0.004 and 0.002, respectively. However, there was no significant difference between K3 and K4 groups. Meanwhile, the statistical analysis showed significant difference between K1 and K3 groups (*P* = 0.041) while the degree of fibrosis between K1 and K4 groups was insignificant.

Macrophage quantification revealed that d‐Galactose‐exposed groups (K2, K3, and K4) had higher number of M1 and M2 than the control (K1) group. Higher number of M1 than M2 was observed in K1 group while the K2 group demonstrated the opposite pattern. The given exercise treatments showed higher number of M1 than M2. The number of M1 in K1 group was significantly lower than in K3 group while the number of M2 in K2 group was significantly higher than in K1 and K3 groups (Table 1). M1/M2 ratio was significantly different. Post hoc test showed that the M1/M2 ratio of K3 and K4 groups was insignificantly different than that of K1.

**Table 1:** Means (SD) of number of M1 and M2 macrophages and M1/M2 ratio of all groups.

Group	*n*	Number of M1/15 FoV	Number of M2/15 FoV	M1/M2 ratio
K1	6	214 (62.59)^a^	138.83 (29.90)^b^	1.55 (0.41)^d^
K2	6	276 (53.08)	307.33 (84.48)^bc^	0.95 (0.27)^de^
K3	6	372.5 (60.06)^a^	206.16 (30.99)^c^	1.87 (0.59)^ef^
K4	6	276 (73.40)	261.83 (79.56)	1.19 (0.63)^f^
*p* Value		*P* = 0.007	*P* = 0.002	*P* = 0.022
		^a^ *P* = 0.017	^b^ *P* = 0.032	^d^ *P* = 0.048
			^c^ *P* = 0.025	^e^ *P* = 0.004
				^f^ *P* = 0.027

*n*, number of animals; *P*, *P* value; K1, 0.9% NaCl intraperitoneal (ip); K2, 300 mg/mL/kg BW d‐Galactose (ip); K3, 300 mg/mL/kg BW d‐Galactose (ip) + low intensity treadmill exercise; K4, 300 mg/mL/kg BW d‐Galactose (ip) + moderate intensity treadmill exercise; FoV, fields of view.

Supercripted letters were used to identify the significant differences between 2 groups. For example, in Number of M1 column, the superscript a letter showed that there is a significant difference between K1 and K3 and the p value is 0.017.

## Discussion

Our study showed the increase of degree of liver fibrosis in d‐Galactose‐exposed aging rat model which could be associated with the increment of M2 macrophage number. Treadmill exercise attenuated the degree of fibrosis and reduced the number of M2. The given moderate exercise might have restored M1/M2 ratio back to the control condition.

Decrease in physiological processes may be caused by aging. Aging occurs throughout the body. It can cause changes in body response, greatly affects liver function, and acts as a major risk factor for chronic diseases (Hung et al. 2010). There are many factors that affect aging including genetic and environmental exposure. In recent years, many studies have discussed the improvement of health and body function in elderly people by physical exercise (Dipietro 2001; Singh 2004).

This study used aging model rats with 300 mg kgBW^−1^ d‐Galactose daily injection for 4 weeks. This dose was chosen following a preliminary study. The results from the preliminary study showed an increase in malondialdehyde (MDA) as much as 3‐5 times of the normal value, elongation of QT interval on ECG results and also proteinuria. The results of this preliminary study show that this dose induced aging. This was in accordance with studies by Mutlu‐Turkoglu et al. (2003) and Gil et al. (2002).

Liver is the largest organ in human body that acts as a lymphoid organ and functions in both adaptive and innate immunity (Bogdanos et al. 2013; Sherwood, 2014). Studies by Hung et al. (2010) and Kim et al. (2015) show that aging can affect liver function by increasing the risk for hepatic injury and making the liver more susceptible to fibrotic response. Hepatic fibrosis is a condition caused by excessive deposition of extracellular matrix and a decrease in matrix metalloproteinase (MMP) production. This condition is caused by continuous hepatocyte injury as a part of normal wound healing responses (Mona and Pinzani, 2009). The degree of fibrosis in d‐Galactose‐exposed groups were significantly higher than that of the control group with the average fibrosis score of four (4), indicating that fibrous expansion of portal areas with marked bridging portal to portal and portal to central was observed. This showed that intraperitoneal administration of d‐Galactose causes fibrosis in liver tissue. This result is in accordance with the study conducted by Ji et al. (2017) that d‐Galactose causes oxidative stress in liver tissue by generating ROS. ROS stimulates the activation and proliferation of myofibroblasts which then produce collagen fiber excess in the tissue (Morry et al. 2016). Increasing production of ROS also leads to lipid peroxidation of cell and mitochondrial membranes, indicated by the production of cytotoxic aldehydes such as MDA which is in line with the result of our preliminary study.

In this study, low and moderate treadmill exercise significantly lowered the degree of fibrosis into average fibrosis score of two (2). These results showed that marked portal to portal and portal to central bridging of collagen fibers was reduced after performing treadmill exercise. There was no significant difference between K1 and K4 groups, indicating that the given moderate intensity treadmill exercise was able to repair the injured liver tissue back to its non‐aging condition. It has been known that exercise can improve the antioxidant system of the body (He et al. 2016). Enhanced antioxidant system can reduce the oxidative stress in the tissue by ROS removal (Nunes‐Silva & Freitas Lima 2014). Albano et al. (2005) and van der Windt et al. (2018) also reported that treadmill exercise reduces markers of fibrosis such as collagen 1α1 mRNA, α‐smooth muscle actin, and fibrosis scores in rats through decreased activation of hepatic stellate cells (HSCs). HSCs and hepatic fibroblasts are the major source of myofibroblasts which are collagen‐producing cells (Lemoinne et al. 2013; Karin et al. 2016). These results are in line with the research conducted by Limongi et al. (2016) reporting that exercise can improve the function of organs, especially the liver.

Hepatic macrophages play an important role in liver disease by activating HSCs and releasing cytokines and pro‐inflammatory chemokines. These cells are crucial in the pathogenesis of hepatic injury and are able to differentiate into various subtypes of alternatively‐activated M1 and classically‐activated M2 macrophages which function in both fibrosis progression and regression. According to Weng et al. (2013), macrophages differentiate into M1 and M2 when fibrosis occurs. The number of macrophages will increase in the event of damage (Heymann et al. 2009; Tacke and Zimmermann, 2014) which is consistent with the results of the present study. The number of M1 and M2 were higher in d‐Galactose‐exposed groups than the control group, indicating that d‐Galactose exposure caused chronic inflammation in liver tissue marked by the elevated number of both macrophage subtypes. These increasing number of macrophages produce cytokines which may cause insulin resistance in adipose tissue, liver and muscles. In K2 group, M2 which plays important roles in tissue repair and are known to secrete molecules contributing to chronic liver inflammation (IL‐10, TGF‐β1, and PDGF) (Wynn and Vannella, 2016) was more predominant in liver tissue than M1. Excessive M2 responses can cause fibrosis (Ju and Mandrekar, 2015) and also its resolution by producing certain kind of matrix metalloproteinase for tissue remodelling (Feng et al. 2018). In line with the degree of fibrosis data, K2 group had the highest fibrosis score which correlates with the high number of M2. Higher number of M1 than M2 was observed in low and moderate treadmill exercise groups. M1 secretes IL‐1 and TNF‐α which can weaken hepatic fibrosis by inducing HSCs apoptosis (Ohtsuki et al. 2016). These results also indicate that treadmill exercise can lower the number of d‐Galactose‐exposed M2. Exercise ameliorates inflammation and fibrosis by decreasing macrophage infiltration in liver tissue (Kawanishi et al. 2012).

The abundance presence of M2 in fibrotic liver protects against hepatocyte apoptosis, hence lethal consequences can be avoided (Bai et al. 2017). This was in line with the results of the present study where rats with the highest fibrosis score in K2 group had a significantly lower M1/M2 ratio compared to the control group. The low ratio was caused by the elevated number of M2 which are responsible for tissue repair and both fibrosis progression and regression. As we took the liver samples 4 weeks after the d‐Galactose induction, the liver might have undergone inflammation resolution and fibrosis regression which are dominated by M2. A study by Troeger et al. (2012) showed that in day 30, roughly half the hepatic stellate cell population was deactivated, indicating fibrosis regression state. The activation of M2 inhibits the activity and induces apoptosis of pro‐inflammatory M1 and eventually leads to the inhibition of hepatic stellate cell activity. However, higher than 1.00 M1/M2 ratios were observed in K3 and K4 groups. This result was not in line with previous studies reporting that exercise induces M2‐polarization, leading to lower M1/M2 ratio. d‐Galactose exposure and exercise are known to increase the expression of PPARγ which mediates the activation of M2 (Goh et al. 2016; Ruffino et al. 2016; Li et al. 2017). It is also possible that higher than 1.00 M1/M2 ratio in both exercise‐treated groups was due to the lower frequency (30 min, 4 times a week) and duration (4 weeks) of the exercise we employed. A study by Jeong et al. (2015) showed that 30‐60 min/day treadmill run 5 times a week for 12 weeks could stimulate M1 shifting into M2 in C57BL/6 mouse liver. Another study reported that a moderate 45‐minute walking session on a treadmill three times a week for 8 weeks supressed M1 markers and induced M2 markers in circulating monocyte of females, resulting in lower M1/M2 ratio (Ruffino et al. 2016). K4 exhibited lower M1/M2 ratio than K1, indicating that the given moderate intensity treadmill exercise has started to induce more M1 to M2 shifting, just like the more frequent and longer duration of exercise from the aforementioned studies. Nevertheless, aging alters the condition of resident cells in tissues, therefore it also affects macrophage polarization (Campbell and Turner, 2018). Gibon et al. (2016) reported that aged M2 from bone marrow lacks of arginase‐1 expression which was the antibody we used to detect M2 in the present study. Other studies also suggest that M1 is activated with the presence of Th1 cytokines such as IFN‐γ or IL‐12 (Ishizuka et al. 2012). Terra et al. (2013) and Zamani et al. (2017) showed that exercise increases IFN‐γ and IL‐12 production, hence the increasing number of M1. Nevertheless, M1/M2 ratios in K3 and K4 groups were insignificant to that of K1 group, indicating that low and moderate exercises might have driven M1/M2 ratio closer to the control state.

## Conclusion

In conclusion, this experiment shows that low and moderate treadmill exercise significantly lower the degree of d‐Galactose‐exposed hepatic fibrosis, and moderate treadmill exercise was able to restore the fibrotic liver tissue back to the non‐aging state. Administration of d‐Galactose causes inflammation marked by the elevated number of M1 and M2 macrophages. Moderate treadmill exercise drove M1/M2 ratio back to the control condition.

## Conflict of Interest

All authors have no conflict of interests with respect to the data collected and procedures used within this study.
